# Broadband photodetector based on carbon nanotube thin film/single layer graphene Schottky junction

**DOI:** 10.1038/srep38569

**Published:** 2016-12-08

**Authors:** Teng-Fei Zhang, Zhi-Peng Li, Jiu-Zhen Wang, Wei-Yu Kong, Guo-An Wu, Yu-Zhen Zheng, Yuan-Wei Zhao, En-Xu Yao, Nai-Xi Zhuang, Lin-Bao Luo

**Affiliations:** 1 School of Electronic Science and Applied Physics, Hefei University of Technology, Hefei, Anhui 230009, P. R. China

## Abstract

In this study, we present a broadband nano-photodetector based on single-layer graphene (SLG)-carbon nanotube thin film (CNTF) Schottky junction. It was found that the as-fabricated device exhibited obvious sensitivity to a wide range of illumination, with peak sensitivity at 600 and 920 nm. In addition, the SLG-CNTF device had a fast response speed (τ_r_ = 68 μs, τ_f_ = 78 μs) and good reproducibility in a wide range of switching frequencies (50–5400 Hz). The on-off ratio, responsivity, and detectivity of the device were estimated to be 1 × 10^2^, 209 mAW^−1^ and 4.87 × 10^10^ cm Hz^1/2^ W^−1^, respectively. What is more, other device parameters including linear performance θ and linear dynamic range (LDR) were calculated to be 0.99 and 58.8 dB, respectively, which were relatively better than other carbon nanotube based devices. The totality of the above study signifies that the present SLG-CNTF Schottky junction broadband nano-photodetector may have promising application in future nano-optoelectronic devices and systems.

In the past several decades, the carbon family including fullerene^1^, carbon nanodots (CQDs)^2^, carbon nanotubes (CNTs)^3^,^4^, graphene^5^, and graphene quantum dots (GQDs)^6^, has attracted tremendous research interest due to its superior and uniquely chemical, optical, physical, mechanical and electronic properties^7^. Take semiconducting single-walled carbon nanotubes (semi-SWCNTs) for example, as one-dimensional (1-D) quantum-confined form of carbon allotropes, semi-SWCNTs are promising building blocks for fabricating high-performance photovoltaic devices because of their near-infrared (NIR) band gaps, strong optical absorptivity (>10^5^ cm^−1^), ultrafast charge transport mobility^8^. What is more, SWCNTs are also promising candidates for future infrared (IR) detectors due to their unique band structure, excellent electronic and optoelectronic properties, and super mechanical and chemical stabilities^9^. To date, a number of IR photodetectors have been successfully demonstrated based on both individual CNT and CNTs films^10^,^11^. Very recently, a prototype infrared camera was developed using single CNT photodetectors^12^. In comparison with devices assembled from other semiconductor materials, the photodetectors based on CNTs have unique advantages in IR detection in terms of ease of construction, low fabrication cost, and scalability. By this token, many different types photodetectors including photodiodes^13^, photoconductors^14^, and bolometers^15^ with high performance are enormously emerging.

Graphene which is a single layer of carbon atoms in a closely packed 2D honeycomb lattice has become one of the most hotly pursued materials since its discovery in 2004. Owing to its fascinating physical properties such as high electrical conductivity (10^8^ S/m), and ultrahigh mobility (200000 cm^−2^/V ∙ s at room temperature), graphene has great potential in fabricating thinner and faster response speed optoelectronic devices^16^,^17^. However, it is undeniable that due to the relatively low absorbance of a single sheet of carbon atoms, graphene-based photodetectors normally suffer from relatively low responsivity. In order to address this issue, a number of semiconductor nanostructures such as II-VI (ZnO^18^,^19^, ZnTe^20^, IV (Si^21^,^22^, Ge^23^, and III-VI (GaN^24^, InAs^25^ have been selected to form Schottky junction based photodiode. These devices exhibit obvious advantages in sensitivity and response speed. What is more, due to the photovoltaic characteristics of the Schottky junction, the majority of the semiconductor-graphene devices are capable of detecting light irradiation without power supply. Enlightened by this, we herein present the fabrication of a high sensitive nano-photodetector by forming a single layer graphene (SLG)-carbon nanotube film (CNTF) Schottky heterojunction. Device performance analysis revealed that the as-fabricated photodetector exhibited high sensitivity to a wide spectrum of light illumination, and it was able to detect very fast optical signal with frequency as high as 5400 Hz, the rise/fall time were estimated to be 68 and 78 μs, respectively. What is more, the on-off ratio, responsivity, and detectivity of the device were calculated to be 10^2^, 209 mAW^−1^ and 4.87 × 10^10^ cmHz^1/2^W^−1^, which are higher than other carbon nanotubes based devices. This study suggests that present SLG-CNTF Schottky junction device will have potential application in future optoelectronic devices.

## Results

**Figure 1:**
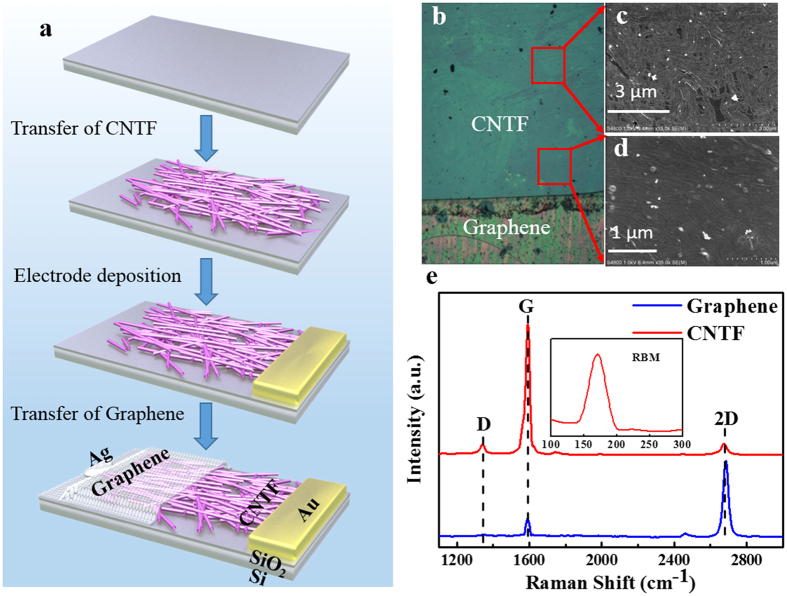
Device fabrication and structural characterization. (**a**) Illustration of the step-wise flow chart for fabrication of SLG-CNTF Schottky junction photodetector. (**b**) Digital camera picture of SLG-CNTF Schottky junction device. (**c**,**d**) SEM images of the CNTF at different magnifications. (**e**) Raman spectra of CNTF (red line) and SLG (blue line), the inset shows the Raman shift corresponding to RBM.

Figure 1a illustrates the detailed procedure for the fabrication of the SLG-CNTF Schottky junction nano-photodetector. Briefly, the fabrication starts with the self-assembly of monolayer CNTF through an Langmuir-Blodgett (LB) approach, during which uniaxial compression of subphase will lead to the self-assembly of carbon nanotube over a large area in a parallel way. The as-assembled monolayer CNTF was transferred on to a SiO_2_/Si wafer, and then partially covered with a SLG film, followed by painting of silver paste on the surface. According to previous study, only gold can forms *Ohmic* contact to the CNTF with negligible contact resistance^26^, therefore, in this study gold film with 50 nm was deposited on the CNTF side. Figure 1b shows a representative microscopy image of the SLG-CNTF interface. It is clear that due to their distinct contrast, both the SLG and CNTF can be easily visualized. Further scanning electron microscopy (SEM) image reveals that the as-assembled CNTF was composed of large-area flexible carbon nanotube array with only single layer [Fig. 1c and d]. To unveil the microstructure of both SLG and CNTF, the Raman spectra of both materials were further analyzed. As shown in Fig. 1e, for the graphene, there are totally three peaks: a 2D peak (2680 cm^−1^) and a G peak (1590 cm^−1^) with an intensity ratio of *I*_2D_:*I*_G_ of 3:1, and a weak D-band scattering at ~1340 cm^−1^, confirming the single-layer of the graphene^27^,^28^. The sheet resistance of the graphene, according to our further electrical study is around 500–800 Ω/□. With regard to the CNTF, similar three peaks can be observed. In addition, there is an obvious signal in the range from 100–300 cm^−1^ which can be readily ascribed to the radial breathing mode (RBM) of single-walled carbon nanotubes^29^.

**Figure 2:**
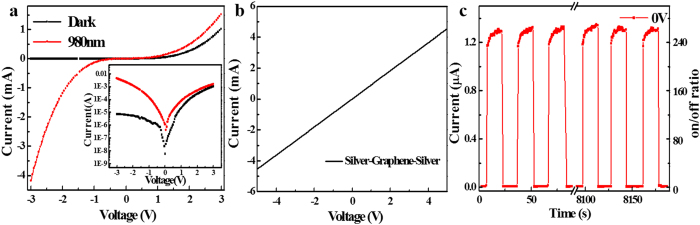
Optoelectronic characteristics of the PD. (**a**) Typical *I-V* characteristics of the CNTF-SLG Schottky junction in dark and under illumination of 980 nm (intensity: 166 mWcm^−2^), the inset shows the *I-V* curve at a log scale. (**b**) *I*-*V* curves of the silver-graphene-silver contact. (**c**) Photoresponse of the photodetector under 980 nm light which is alternately turned on and off at zero bias voltage.

Figure 2a shows a typical room-temperature *I-V* curve of the SLG-CNTF Schottky junction both in dark and under illumination of 980 nm, from which one can observe clearly that the SLG-CNTF interface in dark exhibits typical rectification behavior, with an on/off ratio of ~1 × 10^2^ at ±3 V. In view of the fact that there is not obvious contact resistance between the SLG and silver (Fig. 2b)^30^, and the gold can form good *Ohmic* contact with CNTF^26^, the above rectifying characteristic can be exclusively attributed to the SLG-CNTF interface. In order to quantitatively evaluate the SLG-CNTF Schottky junction, the barrier height was then deduced by using the thermionic-emission based diode equation^31^:


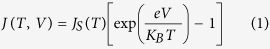


where *J (T, V)* is the current density across the CNTF-SLG interface, *V* the applied voltage *k*_B_ the Boltzmann’s constant, *T* the absolute temperature, *n* the ideality factor 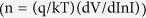, respectively. The prefactor, *Js(T)* is the saturation current density and can be estimated by Js(T) = A*T^2^exp(−*eϕ*_*SBH*_/*K*_*B*_*T*), where *ϕ*_*SBH*_ is the zero bias Schottky barrier height (SBH), A* the Richardson constant, m* the effective mass of the charge carriers, respectively. For CNT, A* is theoretically calculated to be 7.12 × 10^4^ Am^−2^ k^−2^ (m_e_* = 0.06m_0_)^32^, using the *J*_s_ value, the Schottky barrier height at the SLG-CNTF interface was calculated to be 0.78 eV. It is interesting to note that, when irradiated by IR light, such a SLG-CNTF Schottky junction exhibited obvious sensitivity at both negative and forward bias voltage [Fig. 2a]. Figure 2b shows that the switching photoresponse of CNTF-SLG device when the IR irradiation (wavelength: 980 nm, intensity: 166 mWcm^−2^) was switched on and off repeatedly. Apparently, the device can be reproducibly switched between high and low conductance states. The on and off currents were estimated to be 5 nA to 1.2 μA, yielding an on-off ratio as high as 240.

**Figure 3:**
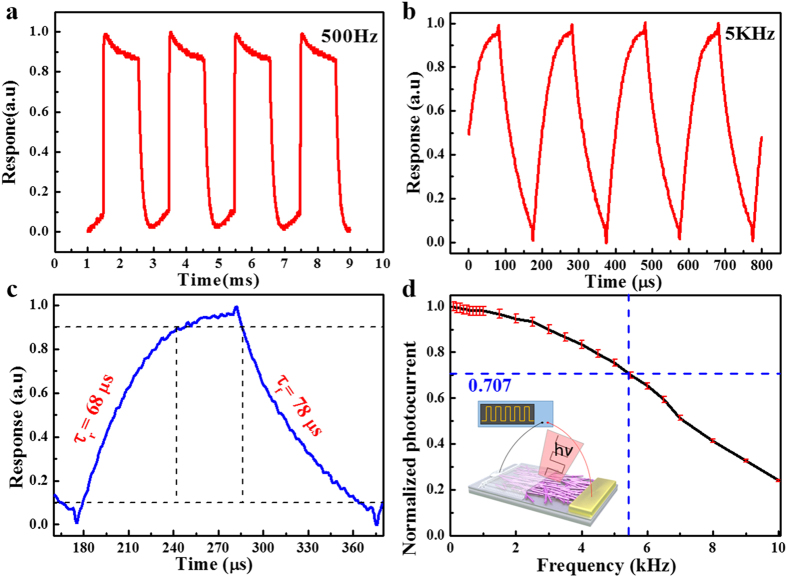
Photoresponse of the PD. (**a**) Photoresponse of the SLG-CNTF device to pulsed infrared irradiation at a frequency of 500 Hz. (**b**) Photoresponse of the CNTF/SLG device to pulsed infrared irradiation at a frequency of 5 kHz. (**c**) One normalized cycle measured at 5 kHz for estimating both rise (τ_r_) and fall time (τ_f_). (**d**) The normalized photocurrent versus switching frequency.

The above photoresponse at zero bias voltage indicated that the SLG-CNTF Schottky junction photodetector can act as a low-consumption device that is capable of detecting IR irradiation without external power supply. To further determine the response rate and explore the feasibility of the present device for practical application in optical switches, the response of the SLG-CNTF Schottky junction photodetector to high-frequency optical signal was studied. During the study, an oscilloscope was used to probe the variation of the photocurrent under pulsed light with varied frequencies. Figure 3a and b depict the photoresponse of the SLG-CNTF device to pulsed IR irradiation at frequency of 500 Hz and 5 kHz, respectively. It can be seen that the device can be alternatively switched between on- and off-state states with excellent reproducibility even under a light pulse with frequency as high as 5 kHz. Further one single cycle of photoresponse under 5 kHz light illumination was plotted in Fig. 3c. By deducing the rise and fall edges, the rise time (*τ*_*r*_, duration for the photocurrent to rise from 10 to 90%) and the fall time (*τ*_*f*_, duration for the photocurrent to decrease from 90 to 10%) were estimated to be 68 and 78 μs, respectively, which are much faster than not only the SWCNT/PCBM heterojunction device (55/37 microseconds), but also than the graphite quantum dots/graphene heterojunction (several seconds)^33^. Understandably, such a fast response speed could be associated with the high-quality Schottky junction formed between the CNTF and SLG, which can facilitate the effective and rapid separation of the photo-generated carriers^34^. According to the normalized photocurrent at different frequencies shown in Fig. 3d, the 3 dB bandwidth is estimated to be 5400 Hz (corresponding to the frequency when the normalized photocurrent decreased from 1 to 0.707), which is much higher than the ZnO/SLG Schottky junction device (<1500 Hz)^18^ and Ge/SLG Schottky junction device (<3000 Hz)^23^, such excellent photoresponse renders the current device a potential building block for future optoelectronic device applications.

**Figure 4:**
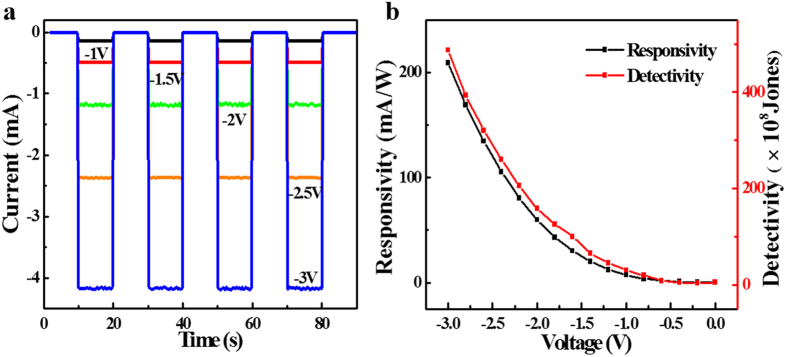
Performance of the PD under different bias voltage. (**a**) Time response of the PD at various bias voltage. (**b**) Responsivity and detectivity of the PD as a function of bias voltage.

In fact, the photosensitivity of our device is highly dependent on the bias voltage. Figure 4a plots the photoresponse under different bias voltage, from which it can be seen that both photocurrent and dark current will increase monotonously with increasing bias voltage. In order to quantitatively assess the bias voltage dependent photoresponse of the SLG-CNTF Schottky junction photodetector, two key device parameters including both responsivity (*R*)^35^ and detectivity (D*)^36^,^37^, were calculated by the following formulas:


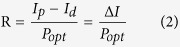



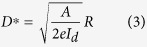


where *I*_p_, *I*_d_, *P*_opt_ and *e* are the photocurrent, the dark current, the power of the light which is irradiated on the device, the elementary charge (1.6 × 10^−19^ Coulombs), respectively. Using above constants and the Equations 2 and 3, the responsivity and detectivity at a bias voltage of −3 V were estimated to be 209 mAW^−1^and 4.87 × 10^10^ cmHz^1/2^W^−1^, respectively. The responsivity and detectivity at different bias voltages were shown in Fig. 4b, in which both parameters were observed to increase with decreasing bias voltage. As matter of fact, these two metrics can be further increased when the bias voltage continues to decrease. Table 1 summarizes the response speed, responsivity and detectivity of the present device and other CNTs based photodetectors. It is obvious that the SLG-CNTF Schottky junction has a high responsivity (209 mAW^−1^) and detectivity (4.87 × 10^10^ cmHz^1/2^W^−1^), which are not only better than the device solely composed of pure SWCNTF^9^ and MWCNTF^11^, but also than the devices assembled from SWCNT/PCBM^38^, and MWCNT/graphene nanocomposite structures^13^.

**Figure 5:**
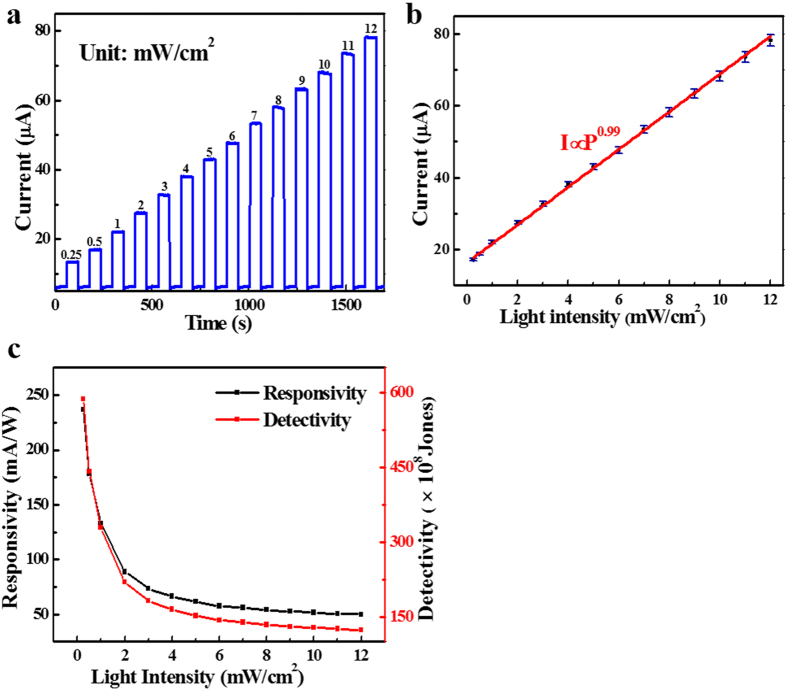
Performance of the PD under different light intensity. (**a**) Time response of the photodetector under 980 nm light with various power intensities. (**b**) The fitting of the relationship between the photocurrent and light intensity. (**c**) Responsivity and detectivity of the photodetector as a function of light intensity.

Further photocurrent study at different light intensities (from 0.25 to 12 mW cm^−2^) reveals that the photocurrent (*I*_p_) of the SLG-CNTF Schottky junction PD exhibits high dependence on the intensity of incident IR. As exhibited in Fig. 5a, the photocurrent of the SLG-CNTF Schottky junction PD increases gradually with increasing intensity. Numerical analysis of the relationship between photocurrent and light intensity reveals that the photocurrent can be described by a simple power law: *I*_*p*_ = *CP*^*θ*^ 39 where *C* is a constant for the IR light, and the exponent *θ* (0.5 < *θ* < 1) determines the sensitivity of photocurrent to the incident IR intensity. By fitting the above formula to the experimental data, *θ* is determined to be 0.99 [Fig. 5b]. Such a nearly integer value suggests a low density of trap states in this SLG-CNTF photodetector. One of the figure-of-merit of the device is the linear dynamic range (LDR, normally quoted in dB) which is given by:


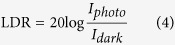


where *I*_photo_ is the photocurrent measured at a light intensity of 1 mW/cm^2^. By using the above equation, the LDR is estimated to be 58.8 dB which is higher than that of the SWCNT/PCBM heterojunction (40 dB)^38^. Such a relatively large LDR suggests the great linear response characteristic of the device. In order to further unveil the light intensity dependent photoresponse of SLG-CNTF device, the responsivity and detectivity of the nano-photodetector in the low range from 0 to 12 mW/cm^2^ were studied. As plotted in Fig. 5c, the responsivity and detectivity will gradually increase with increasing intensity. On the contrary, with further increase of the intensity, the responsivity and detectivity begin to saturate, which is due to the reduced carrier recombination rate at high light intensity^40^.

**Figure 6:**
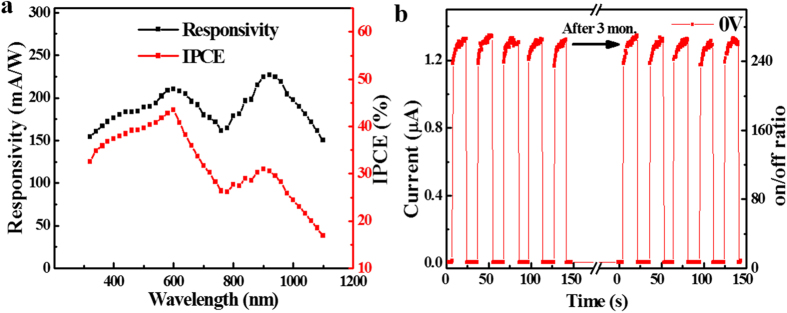
Spectral response and stability of the PD. (**a**) Responsivity and IPCE of the SLG-CNTF as a function of wavelengths. (**b**) Stability of the PD after long-term storage.

Figure 6 plots the responsivity and IPCE of the CNTF-SLG Schottky junction PD as a function of wavelength (To make the analysis more reliable, we kept the light power identical for all wavelengths during testing). Apparently, the SLG-CNTF Schottky junction PD shows a broadband sensitivity: namely it is nearly sensitive to all irradiation in the range from 300–1100 nm. Obviously, such a broadband spectral selectivity is primarily due to the operation mechanism. The generation of the photocurrent in the Schotty junction device is limited to by the electrical property of semiconducting carbon nanotube, whose bandgap is around 1.1 eV, corresponding to 1100 nm in wavelength. When shined by light illumination, the photons with energy larger than the bandgap of carbon nanotube (1.1 eV) can lift the electrons in the valence band to the conduction band, and therefore they can contribute to the photocurrent. In addition, there are two obvious peaks at 600 and 920 nm. Such a spectral selectivity is similar to the incident photon-to-electron conversion efficiency (IPCE), which can be calculated by the following equation


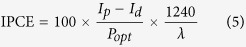


where *λ* is the wavelength of illuminated light (320–1100 nm), *I*_p_ and *I*_d_ are the photocurrent and the dark current, respectively. As indicated by the red curve in Fig. 6a, the device has an IPCE exceeding 20% over a broad range (320–1050 nm), with two maximum values (43% at 600 nm, and 31% at 900 nm). The detailed origin for this phenomenon is still unknown and needs further exploration. In addition, the present PD has very good stability. It can virtually keep the same photocurrent and on/off ratio even the device was stored in ambient condition for 3 months (Fig. 6b).

**Figure 7:**
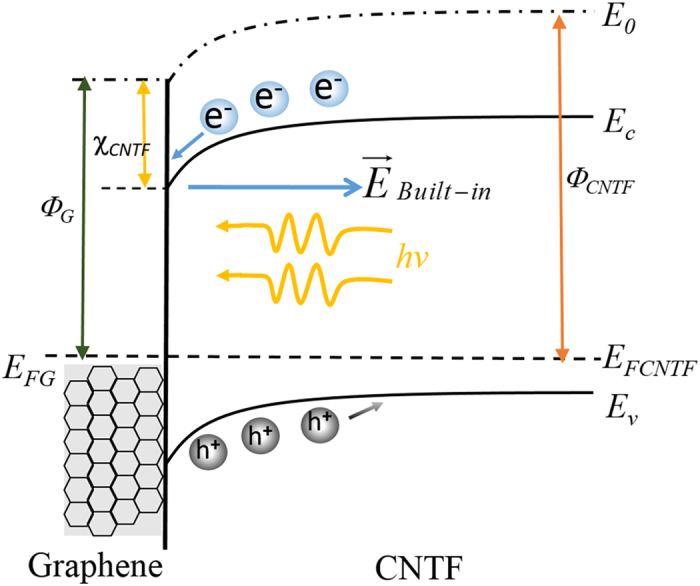
Energy band diagram of the PD. Energy band diagram of the photodetector under light illumination at reverse bias.

The operation of the SLG-CNTF Schottky junction IR photodetector can be interpreted by the energy band diagram illustrated in Fig. 7. When metallic SLG was transferred onto the CNTF, a built-in potential in CNTF near the interface (the depletion region) was formed due to the difference in work functions between the SLG and CNTF. Such a SLG-CNTF interface, like conventional metal-semiconductor junction, can allows the current to flow only in one direction, thus, leading to a rectifying behavior. When irradiated by IR light, the semiconducting CNTF will absorb photons with sufficient energy, and then form huge amount of electron-hole pairs. The photo-induced electrons-holes then drift to the SLG-CNTF interface, and are separated by the built-in electric field. Finally, the electron and holes will be collected by Ag and Au electrode, respectively, leading to the formation of photocurrent in the external circuit. Note that during this detecting process, thanks to the high quality of the SLG-CNTF Schottky junction, the photo-generated electron-holes pairs will be effectively and rapidly separated^41^. As a consequence, the present device is able to detect IR signal with a relatively rapid response.

In conclusion, we have demonstrated a highly sensitive nano-photodetector by coating a layer of SLG onto a CNTF which was obtained by an LB method. Device performance analysis reveals that the as-assembled SLG-CNTF device exhibits high sensitivity to a wide spectrum of light illumination, with two peak sensitivity at 600 and 920 nm, respectively. Specifically, the device was able to probe fast-switching optical signal with frequency as high as 5400 Hz with very good reproducibility. The rise/fall times were estimated to be 68 and 78 μs, respectively. What is more, the on-off ratio, responsivity, and detectivity of the device were calculated to be 10^2^, 209 mAW^−1^ and 4.87 × 10^10^ cmHz^1/2^W^−1^, respectively, which are higher than that of other photodetectors. This study suggests that the current CNTF/SLG Schottky junction broadband photodetector will have potential application in future optoelectronic devices.

## Methods

### Synthesis and Characterization of SLG Film and CNTF

The single layer graphene (SLG) films were fabricated *via* a chemical vapor deposition (CVD) method at around 1000 °C using a mixed gas of CH_4_ (40 standard cubic centimeter per minute, SCCM)) and H_2_ (20 SCCM) as precursors^18^. After deposition, the 25 μm thick Cu foil covered with SLG film was spin-coated with polymethylmethacrylate (PMMA) solution (5 wt% in chlorobenzene) and the Cu substrates were etched away by the Marble’s reagent solution. The mono-layer carbon nanotube array was assembled by using an Langmuir-Blodgett trough (Nima Tech., 312D). Briefly, purified nanotube soot with diameter of 1 nm was firstly dispersed in a mixed solution of trichloromethane and N,N-Dimethylformamide (DMF) with a volume ratio of 1:1. After the trough was filled with distilled water, the resultant solution (100 μL) was then dispensed on the water sub-phase by using a 20 μL syringe drop by drop. The carbon nanotube layer was then compressed carefully until multilayer was formed. Finally, the monolayer nanotube array was loaded onto a silicon SiO_2_ (500 nm)/Si substrate by slowly lifting the substrate from the sub-phase^42^. The Raman study of both SLG and carbon nanotube was performed on a Raman spectrum (JY, LabRAM HR800).

### Device Fabrication and Characterization

To fabricate the CNTF/SLG Schottky junction photodetector, 50 nm Au electrode which served as electrical contact for graphene, was first deposited onto the one side of CNTF on SiO_2_ substrate using E-beam evaporation. Then the PMMA-supported SLG were directly transferred onto the other side of CNTF on SiO_2_ substrate. After drying at 100 °C for 10 min, the residual PMMA on SLG was removed by acetone. The device characteristics of CNTF/SLG were measured using an I-V characterization system (Keithley Company, SCS-4200). To determine the spectral response and time response of the Schottky junction device, a home-built system composed of a light source (Energetiq, EQ99-X), a monochromator (Princeton Instrument, SP2150) and an oscilloscope (Tektronix, DPO 5104B) was used.

## Additional Information

**How to cite this article**: Zhang, T.-F. *et al*. Broadband photodetector based on carbon nanotube thin film/single layer graphene Schottky junction. *Sci. Rep.* **6**, 38569; doi: 10.1038/srep38569 (2016).

**Publisher’s note:** Springer Nature remains neutral with regard to jurisdictional claims in published maps and institutional affiliations.

## Figures and Tables

**Table 1:** Summary of the performance of the present and other CNF based devices.

Materials and structures	τ_r_	τ_f_	*R*[mAW^−1^]	D^*^ [cmHz^1/2^W^−1^]	Ref.
SLG-CNTF	68 μs	78 μs	209	4.87 × 10^10^	Our work
SWCNT/PCBM	55 s	37 μs	20	2 × 10^10^	38
MWCNT/Graphene	~1 ms	~1 ms	—	10^7^	13
SWCNTF	—	—	0.98	10^7^	9
MWCNTF	~1 ms	~1 ms	—	3.3 × 10^6^	11
